# Spatiotemporal prevalence of COVID-19 and SARS-CoV-2 variants in Africa

**DOI:** 10.3389/fpubh.2025.1526727

**Published:** 2025-02-20

**Authors:** Li-Ping Gao, Can-Jun Zheng, Ting-Ting Tian, Alie Brima Tia, Michael K. Abdulai, Kang Xiao, Cao Chen, Dong-Lin Liang, Qi Shi, Zhi-Guo Liu, Xiao-Ping Dong

**Affiliations:** ^1^National Key Laboratory of Intelligent Tracking and Forecasting for Infectious Diseases, NHC Key Laboratory of Medical Virology and Viral Diseases, National Institute for Viral Disease Control and Prevention, Chinese Center for Disease Control and Prevention, Beijing, China; ^2^Chinese Center for Disease Control and Prevention, Beijing, China; ^3^Sierra Leone-China Friendship Biological Safety Laboratory, Freetown, Sierra Leone; ^4^National Key Laboratory of Intelligent Tracking and Forecasting for Infectious Diseases, National Institute for Communicable Disease Control and Prevention, Chinese Center for Disease Control and Prevention, Beijing, China; ^5^Shanghai Institute of Infectious Disease and Biosafety, Shanghai, China

**Keywords:** COVID-19, SARS-CoV-2, variant, health indexes, Africa

## Abstract

**Introduction:**

The coronavirus disease 2019 (COVID-19) pandemic has caused significant public health and socioeconomic crises across Africa; however, the prevalent patterns of COVID-19 and the circulating characteristics of severe acute respiratory syndrome coronavirus 2 (SARS-CoV-2) variants in the continent remain insufficiently documented.

**Methods:**

In this study, national data on case numbers, infection incidences, mortality rates, the circulation of SARS-CoV-2 variants, and key health indexes were collected from various official and professional sources between January 2020 and December 2023 were analyzed with SaTScan and geographically weighted regression (GWR).

**Results:**

The prevalent profiles and circulating features of SARS-CoV-2 across the African continent, including its five regions and all African countries, were analyzed. Four major waves of the epidemic were observed. The first wave was closely associated with the introduction of the early SARS-CoV-2 strain while the subsequent waves were linked to the emergence of specific variants, including variants of concern (VOCs) Alpha, Beta, variants of interest (VOIs) Eta (second wave), VOC Delta (third wave), and VOC Omicron (fourth wave). SaTScan analysis identified four large spatiotemporal clusters that affected various countries. A significant number of countries (50 out of 56) reported their first cases during February 2020 and March 2020, predominantly involving individuals with confirmed cross-continental travel histories, mainly from Europe. In total, 12 distinct SARS-CoV-2 VOCs and VOIs were identified, with the most prevalent being VOCs Omicron, Delta, Beta, Alpha, and VOI Eta. Unlike the dominance of VOC Delta during the third wave and Omicron during the fourth wave, VOC Alpha was relatively rare in the Southern regions but more common in the other four regions. At the same time, Beta predominated in the Southern region and Eta in the Western region during the second wave. Additionally, relatively higher COVID-19 case incidences and mortalities were reported in the Southern and Northern African regions. Spearman rank correlation and geographically weighted regression (GWR) analyses of COVID-19 incidences against health indexes in 52 African countries indicate that countries with higher national health expenditures and better personnel indexes tended to report higher case incidences.

**Discussion:**

This study offers a detailed overview of the COVID-19 pandemic in Africa. Strengthening the capacity of health institutions across African countries is essential for the timely detection of new SARS-CoV-2 variants and, consequently, for preparedness against future COVID-19 pandemics and other potentially infectious disease outbreaks.

## Introduction

Coronavirus disease 2019 (COVID-19) was declared a global pandemic by the World Health Organization (WHO) on 11 March 2020, significantly affecting health systems and socioeconomic conditions across the world (1, 2). Although Africa was the last continent to confront the pandemic, it was particularly vulnerable due to inadequate healthcare systems, significant underfunding, and a heavy burden of other infectious and parasitic diseases (3–5). Despite the tireless efforts by healthcare workers, governments, and communities, the pandemic has caused significant health and socioeconomic crises, straining livelihoods and triggering social upheaval across Africa (6, 7). The pandemic has revealed long-standing structural weaknesses in African healthcare systems (8), highlighting the need for improved preparedness for future health emergencies. For instance, the immense demand for diagnostic testing during the early stages of the pandemic, along with the difficulties in ensuring accurate molecular severe acute respiratory syndrome coronavirus 2 (SARS-CoV-2) diagnosis due to limited laboratory capacity, highlighted critical gaps (9). The SARS-CoV-2 virus has undergone continuous evolution and mutation, resulting in the emergence of numerous variants (10). According to the WHO criteria for variants of concern (VOCs), VOC Alpha (Pango lineage: B.1.1.7) was first documented in the UK in September 2020, VOC Beta (Pango lineage: B.1.351) in South Africa in May 2020, VOC Gamma (Pango lineage: P1) in Brazil in November 2020, and VOC Delta (Pango lineage: B.1.617.2) in India in October 2020 (11). Since 2022, VOC Omicron (Pango lineage: B.1.1.529), identified in multiple countries, particularly in Africa, in November 2021, has become the predominant variant globally due to its higher transmissibility and immune escape capabilities (12). Furthermore, VOC Omicron has undergone rapid evolution, resulting in numerous subvariants and sublineages (13). This study aims to analyze the epidemic dynamics of COVID-19 and the prevalence of SARS-CoV-2 variants across Africa comprehensively. Additionally, it analyzes the relationships between COVID-19 incidence and various health indices across African countries.

## Methods

### Data source, data processing, and wave definition

- Data source: epidemiological data were obtained from public COVID-19 datasets (“Our World in Data”), the World Bank^1^, and the global epidemic data analysis and risk assessment platform.^2^
- Wave definition: the pandemic in Africa exhibited four distinct waves, from February 2020 to September 2020, from October 2020 to April 2021, from May 2021 to October 2021, and from November 2021 to December 2022.
- Data processing: data were processed and analyzed following methodologies described in a previous study (14). SaTScan software version 2.0 (National Cancer Institute, Division of Cancer Prevention, Biometry Branch) was used for spatiotemporal cluster analysis of COVID-19 in Africa, and the log-likelihood ratio (LLR) was calculated. Spearman rank correlation analysis was conducted to explore relationships between COVID-19 incidences and several health indices, including domestic general government health expenditure D as a percentage of general government expenditure (GGHE-D/GGE, %), the current health expenditure (CHE) per capita (US$), physicians/1,000 people, nurses, and midwives/1,000 people. Analyses were performed using the R package (open-source) (15). Additionally, geographically weighted regression (GWR) was applied to account for geographical relationships using the R-package “GWmodel.” The bandwidth was determined using the bw.gwr function, and model parameters were estimated with the gwr. basic function.

## Results

### Epidemiology profile of COVID-19 in Africa

**Figure 1:**
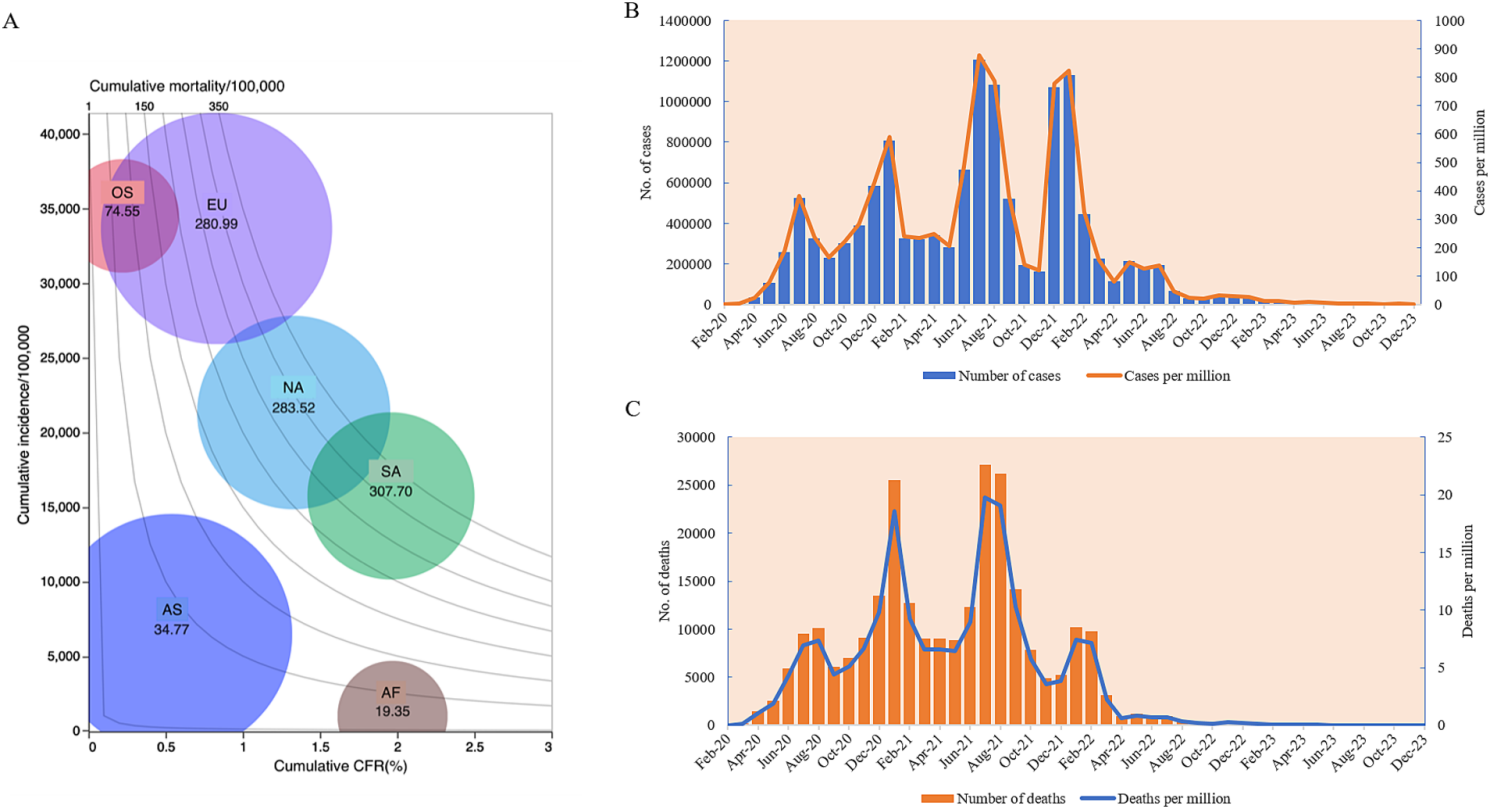
The epidemiological trends of COVID-19 in Africa from January 2020 to December 2023. **(A)** Balloon chart of the cumulative COVID-19 case incidences, mortalities, CFRs, and case numbers of six continents. The *y*-axis represents the incidence per 100,000. The *x*-axis represents CFR (%). Isolines in the contour area represent the mortalities. The size of the balloon indicates the case numbers. AF, Africa; AS, Asia; EU, Europe; NA, North America; SA, South America; OS, Oceania. **(B)** Temporal case numbers (left *y*-axis) and incidences per million (right *y*-axis). **(C)** Temporal death numbers (left *y*-axis), and mortalities per million (right *y*-axis).

As of 31 December 2023, Africa had reported 12,628,665 COVID-19 cases and 258,136 deaths, with a total incidence and mortality of 8,528.44 per million and 174.35 per million, respectively. The crude fatal rate (CFR) was 2.04%. Compared to other continents, Africa exhibited lower cumulative incidence and mortality rates but a higher CFR (Figure 1A). Across the four pandemic waves, confirmed cases numbered 1,482,368 in the 1st wave, 3,075,770 in the second, 3,947,032 in the third, and 4,632,062 in the fourth wave, with the highest case count during the fourth wave (Figure 1B). Deaths totaled 35,898, 85,957, 96,466, and 38,241 across the waves, with the highest death numbers in the third wave (Figure 1C).

### Time and geographic distribution features of cases and deaths in Africa

Monthly, the first two highest case numbers in the context of Africa were reported in July 2021 (*n* = 1,204,828) and January 2022 (*n* = 1,130,918), with 267,616 cases per month on average (Figure 1A). The highest incidence rates were observed in July 2021 (*n* = 877.20/million) and January 2022 (*n* = 823.39/million) (Figure 1B). The highest death numbers were noted in July (*n* = 27,133) and August 2021 (*n* = 26,210), with the highest mortality rate in July 2021 (*n* = 19.75/million) and August 2021(*n* = 19.08/million) (Figure 1C).

**Figure 2:**
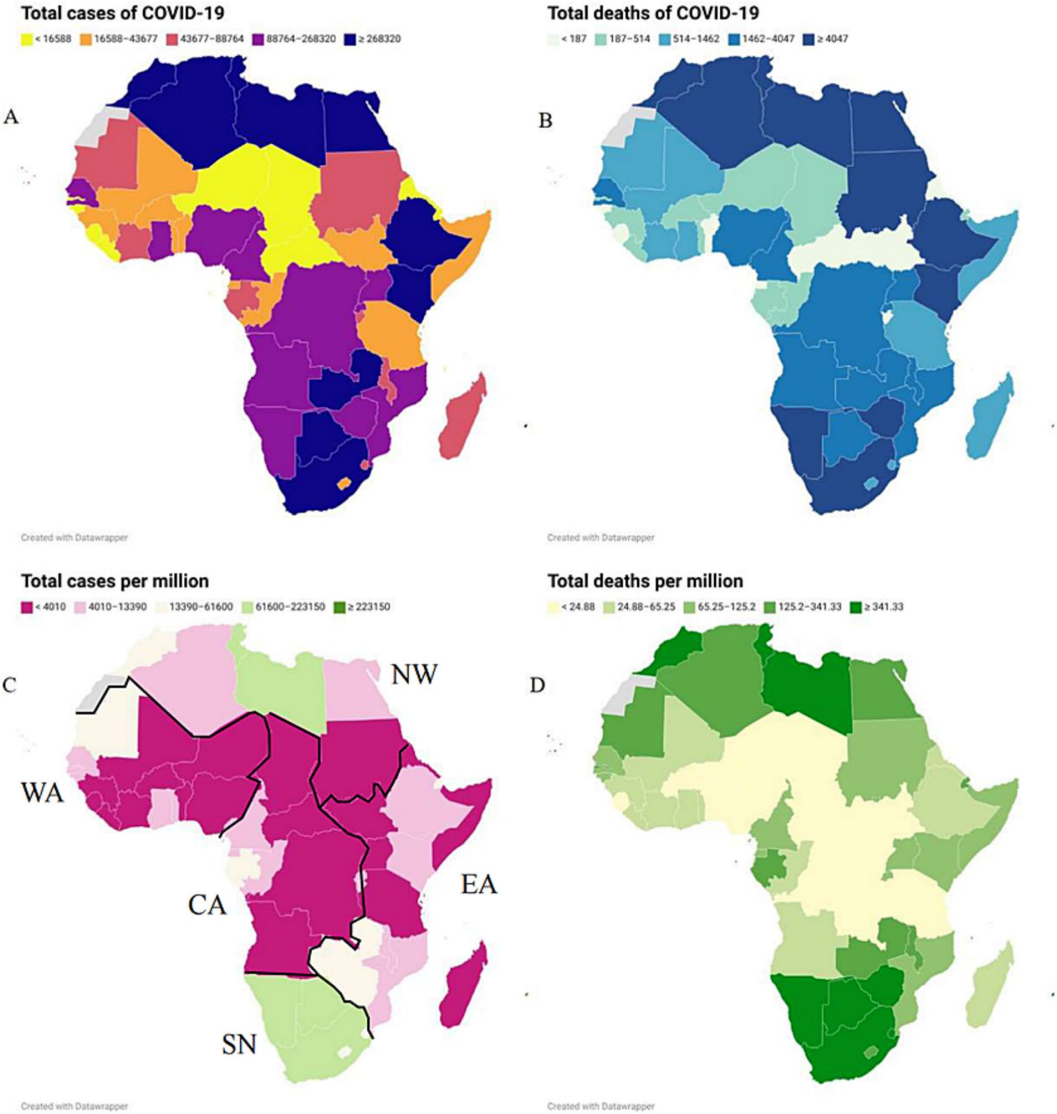
The geographic distributions of the cumulative COVID-19 case number **(A)**, incidence **(B)**, death number **(C)**, mortality **(D)** from January 2020 to December 2023 in African countries.

The case and death numbers of COVID-19 across five African regions and different countries showed significant diversity. The cumulative case and death numbers were 3,809,476 and 89,062 (with a death rate of 2.28%) in 7 countries of Northern Africa; 1,352,325 and 21,049 (with a death rate of 1.53%) in 10 countries in Eastern Africa; 955,678 and 12,182 (with a death rate of 1.26%) in 17 countries of Western Africa; 229,693 and 2,894 (with a death rate of 1.24%) in 8 countries of Central Africa; and 6,281,493 and 132,949 (with a death rate of 2.07%) in 14 countries of Southern Africa, respectively (Figures 2A,B; Table 1). Based on the population numbers of African countries issued at the end of 2023, the cumulative incidence and mortality in Africa were 8,528.44/million and 174.35/million, and the total CFR was 2.04%. The highest incidence (20,635.74 cases/million) and mortality (436.76 cases/million) were observed in the region of Southern Africa, while the lowest incidence (1,680.20/million) and mortality (21.17/million) were reported in Central Africa (Figures 2C,D; Table 1). The total case fatality rate in Africa was 1.27%, in which the top five countries with the highest CFR were Sudan (7.89%), Somalia (4.98%), Egypt (4.81%), Liberia (3.71%), and Niger (3.31%) (Table 1).

**Table 1:** Cumulative cases and death numbers, incidence, mortality and CRF of COVID-19 in African countries.

Region	Country	Total cases	Total deaths	Incidence/million	Mortality/million	CRF
Northern	Algeria	272,010	6,881	6057.69	153.24	2.53%
	Egypt	516,023	24,830	4649.27	223.71	4.81%
	Libya	507,269	6,437	74463.21	944.90	1.27%
	Morocco	1,278,055	16,298	34119.70	435.10	1.28%
	South Sudan	18,765	147	1719.48	13.47	0.78%
	Sudan	63,993	5,046	1365.21	107.65	7.89%
	Tunisia	1,153,361	29,423	93343.33	2381.25	2.55%
	Sum	3,809,476	89,062	13632.30	318.71	2.34%
Eastern	Burundi	54,394	15	4220.00	1.164	0.03%
	Djibouti	15,690	189	13998.29	168.62	1.20%
	Eritrea	10,189	103	2765.71	27.96	1.01%
	Ethiopia	501,117	7,574	4061.58	61.39	1.51%
	Kenya	344,094	5,689	6368.87	105.30	1.65%
	Rwanda	133,208	1,468	9669.08	106.56	1.10%
	Seychelles	51,220	172	478088.39	1605.45	0.34%
	Somalia	27,334	1,361	1553.29	77.34	4.98%
	Tanzania	43,191	846	659.43	12.92	1.96%
	Uganda	171,888	3,632	3637.87	76.87	2.11%
	Sum	1,352,325	21,049	3863.14	61.30	1.56%
Western	Benin	28,036	163	2099.62	12.21	0.58%
	Burkina Faso	22,106	399	974.96	17.60	1.80%
	Cape Verde	64,474	416	108695.43	701.33	0.65%
	Cote d’Ivoire	88,380	835	3138.43	29.65	0.94%
	Gambia	12,626	372	4665.94	137.47	2.95%
	Ghana	171,834	1,462	5133.07	43.67	0.85%
	Guinea	38,572	468	2783.10	33.77	1.21%
	Guinea-Bissau	9,614	177	4565.96	84.06	1.84%
	Liberia	7,930	294	1495.47	55.44	3.71%
	Mali	33,162	743	1467.76	32.89	2.24%
	Mauritania	63,764	997	13463.27	210.51	1.56%
	Niger	9,515	315	363.06	12.02	3.31%
	Nigeria	267,173	3,155	1222.53	14.44	1.18%
	Saint Helena	2,166	0	401036.85	0	0.00%
	Senegal	89,033	1971	5141.53	113.82	2.21%
	Sierra Leone	7,766	125	902.42	14.53	1.61%
	Togo	39,527	290	4466.98	32.77	0.73%
	Sum	955,678	12,182	2142.56	27.31	1.27%
Central	Central African Republic	15,440	113	2767.45	20.25	0.73%
	Chad	7,698	194	434.34	10.95	2.52%
	Comoros	9,109	160	10885.74	191.21	1.76%
	Congo	25,213	389	4222.98	65.15	1.54%
	Democratic Republic of Congo	99,333	1,468	1003.26	14.83	1.48%
	Equatorial Guinea	17,130	183	10227.38	109.26	1.07%
	Gabon	49,051	307	20532.05	128.51	0.63%
	Sao Tome and Principe	6,719	80	29547.96	351.81	1.19%
	Sum	229,693	2,894	1680.20	21.17	1.26%
Southern	Angola	106,303	1937	2986.96	54.43	1.82%
	Botswana	330,409	2,800	125616.47	1064.52	0.85%
	Cameroon	125,136	1974	4482.83	70.72	1.58%
	Eswatini	75,189	1,427	62569.90	1187.50	1.90%
	Lesotho	35,892	709	15565.79	307.48	1.98%
	Madagascar	68,421	1,426	2310.61	48.16	2.08%
	Malawi	89,162	2,686	4369.55	131.63	3.01%
	Mauritius	315,100	1,056	242481.98	812.63	0.34%
	Mayotte	42,027	187	128872.51	573.42	0.44%
	Mozambique	233,731	2,250	7089.31	68.25	0.96%
	Namibia	172,208	4,103	67084.69	1598.35	2.38%
	South Africa	4,072,636	102,595	67997.53	1712.95	2.52%
	Zambia	349,304	4,069	17449.78	203.27	1.16%
	Zimbabwe	265,975	5,730	16296.95	351.09	2.15%
	Sum	6,281,493	132,949	20635.74	436.76	2.12%
Total		12,628,665	258,136	8528.44	174.35	2.04%

CRF, case fatality rate.

### Spatiotemporal scan analysis of the COVID-19 endemic in Africa

**Figure 3:**
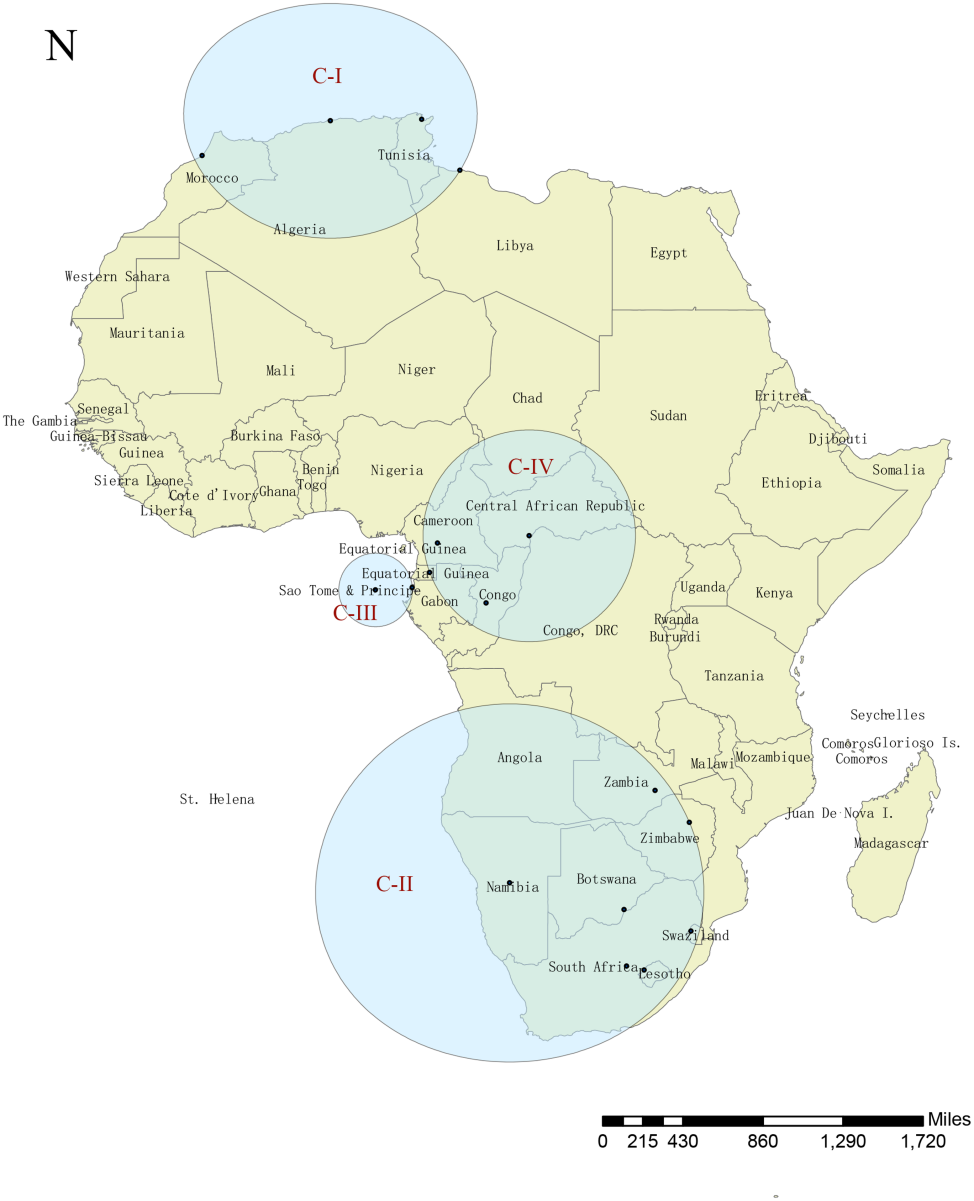
SaTScan analysis for the spatiotemporal clusters of COVID-19 in the African continent.

Four remarkable spatiotemporal clusters were identified, nominated as C-I to C-IV according to the outbreak time (Figure 3; Table 2). C-I occurred from 1 October 2020 to 28 February 2022 (LLR = 2,900,370.90, *p* < 0.05) and covered Tunisia, Algeria, Libya, and Morocco; C-II occurred from 1 December 2020 to 31 March 2022 (LLR = 5,985,737.50, *p* < 0.05), and mainly involved in South Africa, Lesotho, Botswana, Namibia, Eswatini, Zambia, Zimbabwe; C-III involved in Sao Tome and Principe and Gabon and persisted from 1 February 2021 to 31 January 2022 (LLR = 34,407.10, *p* < 0.05); C-IV affected Central African Republic, Cameroon, Equatorial Guinea and Congo from 1 April 2021 to 30 April 2021 (LLR = 10,215.80, *p* < 0.05) (Table 2). The largest and the longest known endemic was C-II, which affected seven countries and lasted for 22 months in Southern Africa.

**Table 2:** SaTScan analysis for COVOD-19 endemic clusters of in Africa.

Clusters	Affected countries	Lasting period	RR	LLR	*p* value
C-I	Tunisia, Algeria, Libya, Morocco	2020/10/1 to 2022/2/28	7.7	2900370.90	<0.01
C-II	South Africa, Lesotho, Botswana, Namibia, Eswatini, Zambia, Zimbabwe	2020/12/1 to 2022/3/31	14	5985737.50	<0.01
C-III	Sao Tome and Principe, Gabon	2021/2/1 to 2022/1/31	5.2	34407.06	<0.01
C-IV	Central African Republic, Cameroon, Equatorial Guinea, Congo	2021/4/1 to 2021/4/30	2.7	10215.77	<0.01

RR, relative risk; LLR, Log likelihood ratio.

### The sources of the first imported COVID-19 cases in various African countries

**Figure 4:**
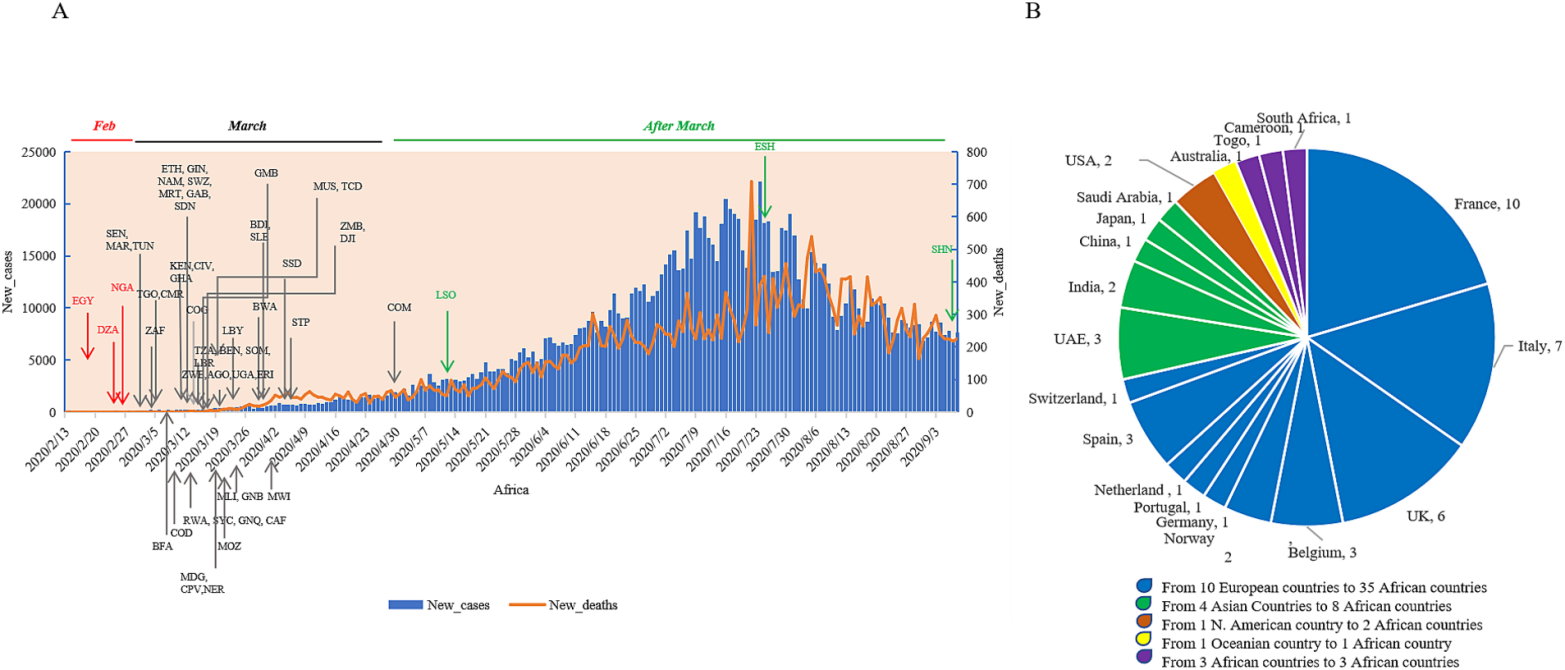
The times and sources of the first confirmed cases of the individual African counties. **(A)** The times of the national first cases. All countries are indicated with the official three-letter acronyms. The daily numbers of new cases (blue column) and new deaths (orange line) are separately indicated in the left and right *y*-axis. **(B)** The sources of the national first cases.

The first confirmed African COVID-19 case was reported in Egypt on 14 February 2020, who was a foreign traveler (16). Afterward, Algeria and Nigeria reported their first COVID-19 case in February 2020 (17, 18). A total of 47 out of 56 African countries reported their first confirmed COVID-19 case in March 2020—3 countries in April 2020, and 1 in May 2020, July 2020, and September 2020, respectively (Figure 4A; Supplementary Table S1). A total of 49 countries described the definite epidemiological sources for their first COVID-19 cases (Supplementary Table S1), closely associated with travel history. Analyzing the geographic sources revealed that the initial cases in 35 African countries and regions originated from 10 European countries, among these 8 African countries traced their cases back to 4 Asian countries, which of 2 African countries were linked to the USA, and 1 to Australia. Furthermore, the initial cases in three countries originated from three other African countries (Figure 4B). France, Italy, and the UK were evidently more relatable, as the first cases from the nine African countries were from France, eight from Italy, six from the UK. A total of 10 African countries reported their first cases before 10 March 2020. All cases were clearly linked to the residence and travel in European countries, indicating that the primary sources for the earliest COVID-19 cases in Africa were from Europe at the start of the pandemic.

### Diversity and similarity in the prevalent profiles of SARS-CoV-2 variants in Africa

**Figure 5:**
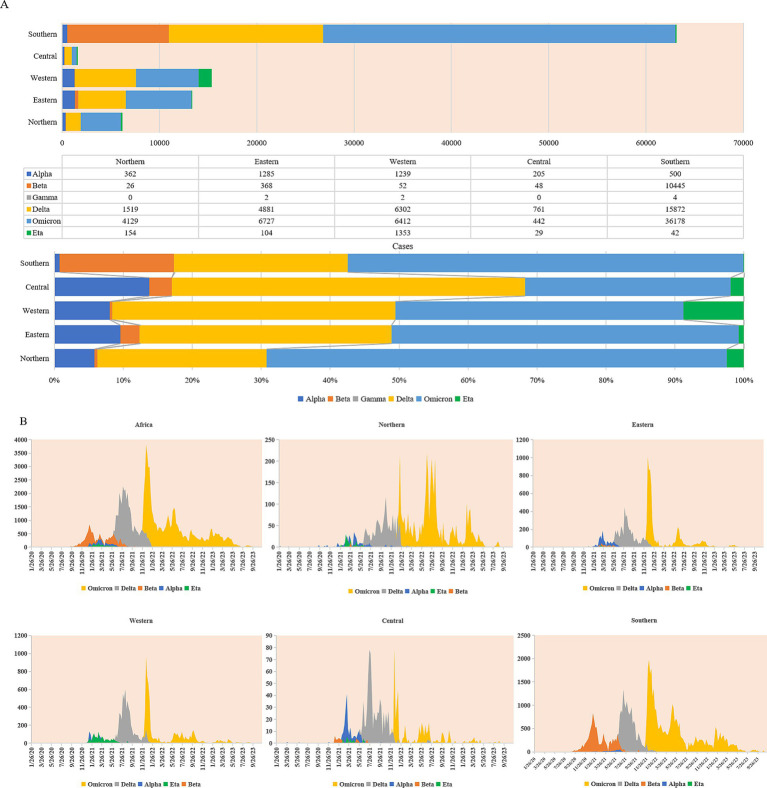
Prevalent profiles of SARS-CoV-2 variants (VOC and VOI) in five African regions. **(A)** Cumulative compositions in case numbers (upper panel) and in percentages (lower panel) of SARS-CoV-2 VOCs (Alpha, Beta, Gamma, Delta, and Omicron) and VOI (Eta). **(B)** Temporal distributing patterns of SARS-CoV-2 VOCs (Alpha, Beta, Gamma, Delta, and Omicron) and VOI (Eta).

Till 31 December 2023, a total of 171,310 cases were confirmed to be infected with various SARS-CoV-2 variants in the African continent. Noticeably, more cases were reported in the countries of Southern Africa, particularly South Africa (Figure 5A; Supplementary Table S2). In terms of VOC or VOI, 12 variants were detected, including VOC Alpha, Beta, Gamma, Delta and Omicron, VOI Epsilon, Zeta, Eta, Theta, Iota, Kappa, and Mu. In the context of the African continent, various VOC and VOI variants of SARS-CoV-2 emerged around the middle of 2020, which is closely associated with the second, third, and fourth waves of endemics. Remarkably, more cases of VOCs (*n* = 127,420) were identified than those of VOIs (*n* = 3,403) (Supplementary Table S2). When considering VOCs, Omicron (56.20%) and Delta (33.60%) were the most frequent, followed by Beta (8.30%) and Alpha (1.90%), while Gamma was rarely detected (only 8 cases). In VOIs, Eta had an overwhelming predominance (97.60%), whereas the other six VOIs were much rare (Supplementary Table S2). The distributions and proportions of the five variants with large case numbers showed obvious diversity (Alpha, Beta, and Eta) and similarity (Delta and Omicron) among the five regions. As shown in Figure 5A, VOC Beta, which was first identified in South Africa, occupied a notably higher portion in Southern Africa, less in Central and Eastern regions, and least in Northern and Western regions. VOI Eta, which was first detected in Nigeria, was most frequently observed in Western Africa, followed by Northern and Central regions, and relatively rare in Eastern and Southern regions. VOC Alpha is distributed in relatively high proportions in Northern, Eastern, Western, and Central regions but remarkably low in Southern Africa. On the contrary, VOC Delta and Omicron occupied comparable proportions among the five regions.

Based on the sampling times, the cases infected with VOC Alpha, Beta, Delta, Omicron, and VOI Eta in the African continent and in the five regions were further analyzed with 7-day average. As shown in Figure 5B, SARS-CoV-2 variants displayed the apparent diversity in the second wave and the similarity in the subsequent third and fourth waves. Variants Alpha, Beta, and Eta were mostly detectable and were responsible for the second wave of endemic. VOC Alpha constantly increased since September 2020 and reached the top around the middle of March 2021, particularly in the Northern, Eastern, and Central regions. VOC Beta has also been clearly detectable since September 2020 and reached the top in the middle of January 2021, mainly concentrated in the Southern region. VOI Eta was observable since Dec 2020 and reached to the top in March 2021, which was most prevalent in Western Africa. The prevalences of variants Alpha, Beta, and Eta in Africa were replaced by the emergence of VOC Delta in May 2021. VOC Delta rapidly reached the top during July 2021 and August 2021 and persisted in December 2021 and January 2022 till the surge of VOC Omicron that was first observed in November 2021 in South Africa and Botswana. VOC Omicron spread quickly in the African continent within a couple of weeks and formed a sharp surge in all five regions. Apparently, the prevalent profiles of VOCs Delta and Omicron were quite similar in the five regions, which were overwhelmingly predominant during the third and fourth waves of endemics.

### Association of COVID-19 incidence with several health indexes

**Figure 6:**
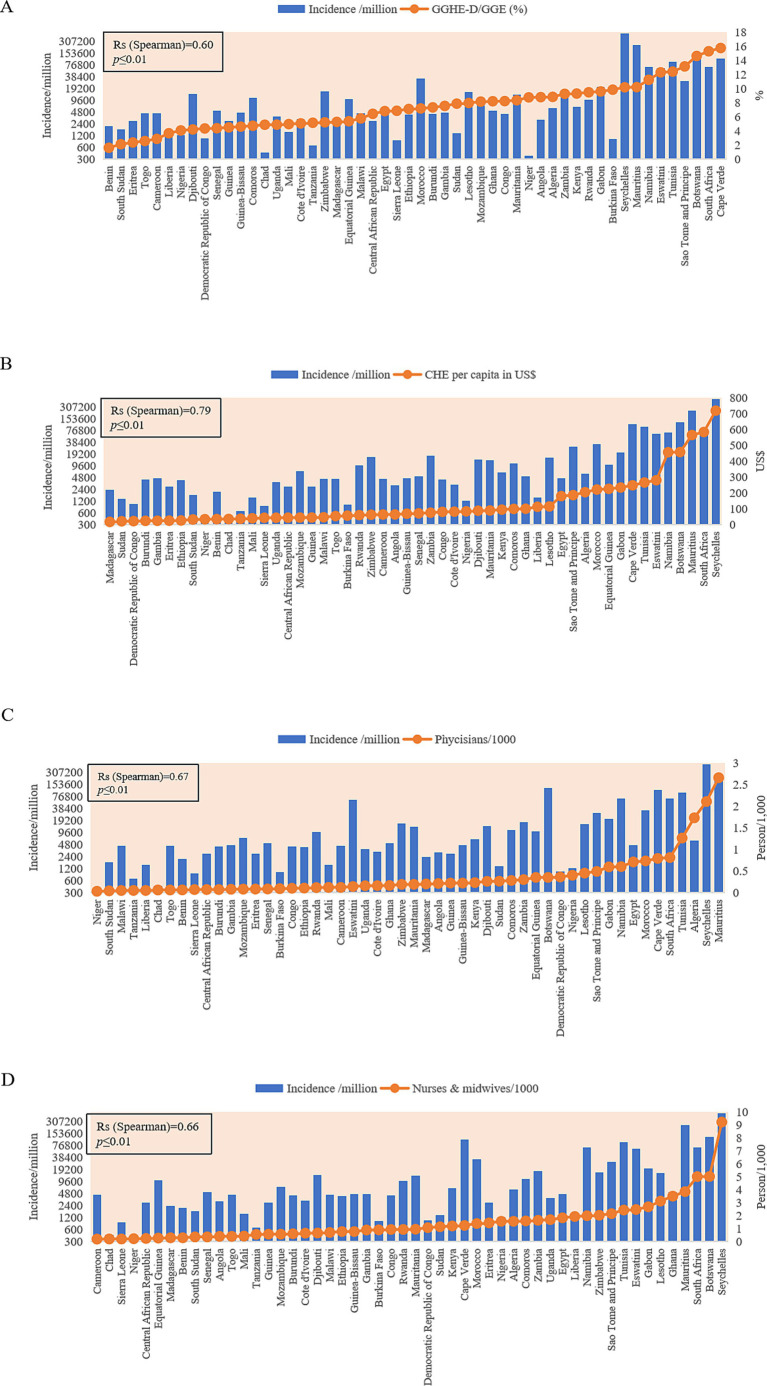
Associations of the cumulative national case incidences (blue column) with four national health indexes (orange dot). **(A)** GGHE-D/GGE (%). **(B)** GHE per capita (US$). **(C)** Physicians per 1,000. **(D)** Nurses and midwives per 1,000. The *y*-axis represents the case incidences per million (in log_2_). GGHE-D, domestic general government health expenditure; GGE, general government expenditure; CHE, current health expenditure.

Four medical indexes of 52 African countries were collected from the database of the World Bank, including the domestic general government health expenditure (GGHE-D) as a percentage of general government expenditure (GGE), the current health expenditure (CHE) per capita in US$, physicians per 1,000 people, and nurses and midwives per 1,000 people (Supplementary Table S3). These elements reflect the investment and capacity of national health. Based on the cumulative case incidences, 52 countries were grouped into low incidence (<2,000 cases/million, 11 countries), middle–low incidence (2,001–6,000 cases/million, 19 countries), middle–high incidence (6,000–20,000 cases/million, 11 countries), and high incidence (>20,001 cases/million, 11countries). The median value of each factor in each group is summarized in Table 3. As expected, the medians of the mortality rates increased step by step from the group of low to high incidence. Apparently, the median values of two elements of the national health expenditures (GGHE-D/GGE (%) and CHE per capita (US$)), and two factors of the national health personals (physicians/1,000, and nurses and midwives/1,000) also showed the increased trend from the group of low incidences to that of high incidence. Further analyses of the individual COVID-19 incidences in 52 countries identified positive associations with health factors. Countries with higher values of GGHE-D/GGE (>10%), CHE per capital (>200 US$), physicians/1,000 (>0.8), or nurses and midwives/1,000 (>2.0) reported higher cumulative COVID-19 incidences, such as Seychelles, Botswana, Mauritius, South Africa, Tunisia, Namibia, and so on (Figure 6). Spearman rank correlation analysis showed that the national case incidence was significantly correlated with GGHE-D/GGE (%), CHE per capita (US$), physicians/1,000, and nurses and midwives/1,000, with the correlation coefficient [Indian National Rupee (INR)] of 0.60, 0.79, 0.67 and 0.66, respectively (Figure 6). Incorporating incidence, GGHE-D/GGE (%), CHE per capita (US $), physicians/1,000, nurses, and midwives/1,000 into the GWR model, the adjusted Akaike information criterion (AICc) is lower than the ordinary least square (OLS), showing regional heterogeneity. And the median of GWR coefficients is 5206.81 (698.44, 69400.40), 243.72 (114.03, 663.41), 111,072 (102,960, 118437.40), and 20983.50(4091.40, 56494.70), respectively, showing a positive correlation, which is consistent with Spearman rank correlation analysis.

**Table 3:** Associations of the cumulative case incidence and mortality with several national health indexes.

	Low incidence (<2,000/million)	Middle-low incidence (2,001–6,000/million)	Middle-high incidence (6,001–20,000/million)	High incidence (>20,000/million)
Country No.	11	19	11	11
Median of incidence (min-max)	1003.26 (363.06–1719.48)	4219.99 (2099.62–5141.53)	10885.74 (6057.69–17449.78)	67997.53 (20532.05–478088.39)
Median of mortality (min-max)	14.53 (10.95–107.65)	54.43 (1.16–223.71)	168.62 (68.25–351.09)	1064.52 (128.51–2381.25)
Median of GGHE-D/GGE (%) (min-max)	4.96 (2.11–9.84)	5.27 (1.60–8.78)	8.15 (4.20–9.47)	12.3 (7.18–15.75)
Median of CHE per capita in US$ (min-max)	37.16 (21.58–112.27)	46.56 (17.64–179.68)	89.29 (44.52–225.57)	279.91 (186.1–718.49)
Median of physicians/1000 (min-max)	0.07 (0.03–0.36)	0.10 (0.07–0.71)	0.26 (0.08–1.73)	0.73 (0.35–2.66)
Median of nurse & midwives/1000 (min-max)	0.55 (0.20–1.93)	0.65 (0.19–3.50)	1.20 (0.57–2.03)	2.47 (1.24–9.22)

GGHE-D, domestic general government health expenditure.

GGE, general government expenditure.

CHE, current health expenditure.

## Discussion

This study comprehensively analyzed the spatiotemporal epidemiologic characteristics of the COVID-19 pandemic across Africa, examining the entire continent, its five geographic regions, and individual countries. There are four waves of COVID-19 endemics in the African continent, which are closely associated with the prevalences of SARS-CoV-2 early strains (the first wave), VOCs Beta and Alpha, VOI Eta (the second wave), VOC Delta (the third wave), and VOC Omicron (the fourth wave). Apparently, those four large waves are induced by the importation of SARS-CoV-2 early strain and emergencies or importations of new VOCs and/or VOI. Our data here has revealed that the first reported COVID-19 cases in the overwhelming most African countries have had the epidemiological information of cross-continent traveling. The earliest imported cases were identified in Egypt, Algeria, and Nigeria in the mid-to-late February 2020, and quickly, most countries reported their first cases with clear cross-continent traveling histories in March 2020. Multiple importations and introductions of SARS-CoV-2 from other continents to African countries via cross-continent traveling constitute the main prevalent characteristics of COVID-19 in the first 2 months, which immediately trigger the local transmission. It is understandable that European countries are the primary source for the importation of SARS-CoV-2, given that COVID-19 had already spread widely in Europe at that time, particularly in Italy, France, the UK, and so on (19). Closer geographic proximity and increased human interaction have made it easier and more frequent for outbreaks to spread between African and European countries (20, 21). The spread of SARS-CoV-2 in African countries has accelerated rapidly as the epidemic has progressed, with South Africa, Kenya, and Nigeria identified as major sources of virus transmission to other African countries (22). Since April 2020, local transmission has become the primary factor through land travel, population movement, immigration, and so on (23), leading to the confirmed cases reported in almost all countries or regions, ultimately exceeding 5,000 at the end of May. The rapid spread of COVID-19 is described either intra- or internationally, for example, the VOC Omicron-induced epidemic in Gauteng, South Africa, spread to neighboring provinces and major transport nodes after it became the dominant variant (24). The epidemic in major cities plays a more important role in the spread of COVID-19 (25). A study in the United States has confirmed that higher risks of COVID-19 clustering and incidence are consistently observed in metropolitan counties compared to rural ones—those closest to core airports, the most populous counties, and those with the highest proportion of cultural/ethnic minorities (26).

As the pandemic progressed, at least 12 VOCs and VOIs have been identified in the African continent (14), showing different characteristics in transmissibility, virulence, and immune evasion (27, 28). Among them, VOCs Omicron, Delta, Beta, Alpha, and VOI Eta are most detectable. The prevalence of those VOCs and VOIs in five African regions and various countries reveals the great diversity in the second wave and the similarity in the third and fourth waves. VOC alpha, Beta, and VOI Eta are the main SARS-CoV-2 variants for the second wave. VOC alpha, which originated in the UK formed a small peak between December 2020 and May 2021, mainly affecting the countries in Eastern, Central, Northern, and Western regions but much less in the Southern region. VOC Beta, which originated in South Africa, surged mainly in the countries of Southern Africa from September 2020 to August 2021. VOI Eta, which originated in Nigeria, apparently circulated in Western Africa from January 2021 to July 2021. The circulations of those three types of SARS-CoV-2 variants in Africa have quickly vanished since the importation and transmission of VOC Delta in the late spring of 2021, and the latter has been replaced by the emergence of VOC Omicron since the beginning of 2022. Compared to the early variants, VOCs Delta and Omicron display remarkable advantages in terms of transmissibility (29, 30), which make them the single SARS-CoV-2 variant in the third and further waves.

In general, both cumulative incidence and mortality of COVID-19 in Africa are low but CFR is high compared to the other regions of the world (10). Later seroprevalence surveys and postmortem surveillance suggest that the actual number of COVID-19 infections and deaths is likely higher (31, 32). It is well known that preexisting comorbidities account for the severe clinical outcome of COVID-19 patients (33). A high prevalence of comorbid conditions has been reported among the COVID-19 individuals admitted to public hospitals in South Africa, such as previously undiagnosed hypertension, diabetes, HIV and active TB, and some poorly controlled chronic diseases (34). Many factors are also assumed to be related to the incidence and mortality of COVID-19, such as limited testing capacity, poor health infrastructure, and inadequate surveillance systems (35, 36), as well as the environmental co-factor, such as high temperature (37). Analyses of the COVID-19 incidences in 52 African countries with some health indexes in this study have verified close associations that the countries with higher national health expenditures and better indexes of medical personnel usually display higher case incidences. It strongly indicates that a good national medical and health service system benefits to identify COIVD-19 cases timely. Certainly, the national economic situations, population compositions, social mobilization mechanisms, and so on can definitely affect the prevalence and clinical outcome of COVID-19 (38). Further improvement of health capacities in low-income countries will be one of the essential elements for the pandemic preparedness for COVID-19 and other infectious diseases globally (39). Continually strengthening the capacity building of public health institutions in African countries, such as implementation of next-generation sequencing (NGS) and establishment of wastewater-based genome monitoring (40), is crucial for timely and accrual detection of the new variants of SARS-CoV-2, particularly those that show increased human transmission, severe clinical outcome, and compromised efficacy of antiviral therapeutics, vaccines and diagnostic assays, such as RT-qPCR assays in detecting SARS-CoV-2 Omicron sub-lineages (41).

## Limitations

This study offers new insights in understanding the spatiotemporal prevalence of COVID-19 and the evolution profile of SARS-CoV-2 variants in Africa. However, several limitations warrant consideration. First, the analysis relies on publicly available data, which may partially reflect the true epidemic situation due to the socioeconomic disparities among African countries, such as, incomplete reporting and variations in detection rates, mortality, and other related indicators across countries. Second, discontinuous sample testing and limited genomic surveillance in some countries are limits the exploration of the correlations between genomic data and the outcome of patients clinically; third, the socioeconomic and health system correlations may not fully account for other contextual factors, such as cultural or political influences, which restrict broader applicability; caution should be exercised when interpreting the findings of this study.

## Conclusion

Socioeconomic disparities lead to significant heterogeneity in COVID-19 epidemiology across African countries. Countries with stronger healthcare systems often reported higher case incidences, highlighting the significance of a robust healthcare infrastructure for pandemic detection and response. This study highlights the progression of the African pandemic, from initial importation to local transmission, and offers insights into the challenges and opportunities for enhancing pandemic control in the region. Strengthening public health capacities, particularly in resource-constrained environments, remains vital for future pandemic preparedness.

## Data Availability

The original contributions presented in the study are included in the article/Supplementary material, further inquiries can be directed to the corresponding authors.

## Footnotes

^1^ www.data.worldbank.org

^2^ www.epiworld.chinacdc.cn
