# Supplementary material for: Spatiotemporal prevalence of COVID-19 and SARS-CoV-2 variants in Africa
Source: Front Public Health. 2025 Feb 20;13:1526727. doi: 10.3389/fpubh.2025.1526727 (PMC11882590; doi:10.3389/fpubh.2025.1526727)
Supplement: Supplementary file 1 [file Data_Sheet_1.docx]

Supplemental Table 1. The first COVID 19 case in various Africa countries

| Region | Country | Reporting time for 1^st^ case | Geographic source |
| --- | --- | --- | --- |
| Northern | Algeria | Feb 25 | Italy |
|  | Egypt | Feb 14 | - |
|  | Libya | March 24 | Saudi Arabia |
|  | Morocco | March 2 | Italy |
|  | South Sudan | Apr 5 | Netherland |
|  | Sudan | March 13 | United Arab Emirates |
|  | Tunisia | March 2 | Italy |
| Eastern | Burundi | March 30 | United Arab Emirates |
|  | Djibouti | March 18 | Spain |
|  | Eritrea | March 21 | Norway |
|  | Ethiopia | March 13 | Japan |
|  | Kenya | March 12 | USA |
|  | Rwanda | March 14 | India |
|  | Seychelles | March 14 | Italy |
|  | Somalia | March 16 | China |
|  | Tanzania | March 16 | Belgium |
|  | Uganda | March 21 | United Arab Emirates |
| Western | Benin | March 16 | Belgium |
|  | Burkina Faso | March 9 | France |
|  | Cape Verde | March 20 | UK |
|  | Cote d'Ivoire | March 12 | Italy |
|  | Gambia | March 17 | UK |
|  | Ghana | March 12 | Norway, Turkey |
|  | Guinea | March 13 | Belgium, France |
|  | Guinea-Bissau | March 25 | unknown |
|  | Liberia | March 16 | Switzerland |
|  | Mali | March 25 | France |
|  | Mauritania | March 13 | Australia |
|  | Niger | March 20 | Togo, Ghana, Cote d’Ivoire, Burkina Faso |
|  | Nigeria | Feb 27 | Italy |
|  | Saint Helena | Sept 7 | unknown |
|  | Senegal | March 2 | France |
|  | Sierra Leone | March 31 | France |
|  | Togo | March 6 | Benin, Germany, France, Turkey |
| Central | Central African Republic | March 14 | France |
|  | Chad | March 19 | Cameroon |
|  | Comoros | Apr 30 | unknown |
|  | Congo | March 15 | France |
|  | Democratic Republic of Congo | Mach 10 | France |
|  | Equatorial Guinea | March 14 | Spain |
|  | Gabon | March 13 | France |
|  | Sao Tome and Principe | Apr 6 | unknown |
| Southern | Angola | March 21 | Portugal |
|  | Botswana | March 30 | UK |
|  | Cameroon | March 6 | France |
|  | Eswatini | March 13 | USA |
|  | Lesotho | May 13 | South Africa, Saudi Arabia |
|  | Madagascar | March 20 | unclear |
|  | Malawi | Apr 2 | India |
|  | Mauritius | March 19 | UK |
|  | Mayotte | - | - |
|  | Mozambique | March 22 | UK |
|  | Namibia | March 13 | Spain |
|  | South Africa | March 5 | Italy |
|  | Zambia | March 18 | France |
|  | Zimbabwe | March 21 | UK |

Supplemental Table 2. Distribution of various SARS-CoV-2 variants (VOC and VOI) in different Africa countries

| Region | Country | Seq. No. | Seq. Ratio (%) | Alpha | Beta | Gamma | Delta | Omicron | Eta | Epsilon | Iota | Kappa | Lambda | Mu | Theta | Zeta | others |
| --- | --- | --- | --- | --- | --- | --- | --- | --- | --- | --- | --- | --- | --- | --- | --- | --- | --- |
| Northern | Algeria | 774 | 0.28 | 34 | 0 | 0 | 109 | 368 | 14 | 0 | 0 | 0 | 0 | 0 | 0 | 0 | 249 |
|  | Egypt | 4013 | 0.77 | 10 | 0 | 0 | 435 | 2325 | 0 | 0 | 0 | 0 | 0 | 0 | 0 | 0 | 1243 |
|  | Libya | 107 | 0.02 | 9 | 0 | 0 | 4 | 31 | 39 | 0 | 0 | 0 | 0 | 0 | 0 | 0 | 24 |
|  | Morocco | 1814 | 0.14 | 127 | 5 | 0 | 234 | 1031 | 3 | 0 | 0 | 1 | 0 | 4 | 0 | 0 | 409 |
|  | South Sudan | 165 | 0.87 | 1 | 2 | 0 | 80 | 28 | 43 | 0 | 0 | 0 | 0 | 0 | 0 | 0 | 11 |
|  | Sudan | 305 | 0.47 | 6 | 15 | 0 | 95 | 69 | 40 | 0 | 0 | 0 | 0 | 0 | 0 | 0 | 80 |
|  | Tunisia | 1551 | 0.13 | 175 | 4 | 0 | 562 | 277 | 15 | 0 | 0 | 0 | 0 | 0 | 0 | 5 | 513 |
|  | ***Sum*** | ***8729*** |  | ***362*** | ***26*** | ***0*** | ***1519*** | ***4129*** | ***154*** | ***0*** | ***0*** | ***1*** | ***0*** | ***4*** | ***0*** | ***5*** | ***2529*** |
| Eastern | Burundi | 157 | 0.28 | 1 | 5 | 0 | 57 | 69 | 0 | 0 | 0 | 0 | 0 | 0 | 0 | 0 | 80 |
|  | Djibouti | 837 | 5.33 | 76 | 5 | 0 | 55 | 517 | 1 | 0 | 0 | 0 | 0 | 0 | 0 | 0 | 183 |
|  | Eritrea | ND |  |  |  |  |  |  |  |  |  |  |  |  |  |  |  |
|  | Ethiopia | 626 | 0.12 | 27 | 2 | 1 | 422 | 107 |  | 0 | 0 | 0 | 0 | 0 | 0 | 0 | 67 |
|  | Kenya | 12070 | 3.50 | 1144 | 250 | 0 | 2693 | 4674 | 61 | 2 | 0 | 6 | 0 | 0 | 0 | 0 | 3240 |
|  | Rwanda | 740 | 0.55 | 9 | 45 | 0 | 272 | 85 | 5 | 0 | 0 | 0 | 0 | 0 | 0 | 0 | 324 |
|  | Seychelles | 1494 | 2.92 | 5 | 29 | 1 | 821 | 578 | 0 | 0 | 0 | 1 | 0 | 0 | 0 | 0 | 59 |
|  | Somalia | 50 | 0.18 | 7 | 4 | 0 | 7 | 7 | 0 | 0 | 0 | 0 | 0 | 0 | 0 | 0 | 25 |
|  | Tanzania | 29 | 0.06 | 0 | 18 | 0 | 4 | 2 | 0 | 0 | 0 | 0 | 0 | 0 | 0 | 0 | 5 |
|  | Uganda | 1844 | 1.07 | 16 | 10 | 0 | 550 | 688 | 37 | 0 | 0 | 1 | 0 | 0 | 0 | 0 | 409 |
|  | ***Sum*** | ***17847*** |  | ***1285*** | ***368*** | ***2*** | ***4881*** | ***6727*** | ***104*** | ***0*** | ***0*** | ***8*** | ***0*** | ***0*** | ***0*** | ***0*** | ***4392*** |
| Western | Benin | 864 | 3.08 | 66 | 2 | 0 | 224 | 86 | 221 | 0 | 0 | 0 | 0 | 0 | 0 | 0 | 265 |
|  | Burkina Faso | 660 | 2.98 | 4 | 0 | 0 | 47 | 15 | 26 | 0 | 0 | 0 | 0 | 0 | 0 | 0 | 568 |
|  | Cape Verde | 950 | 1.47 | 20 | 0 | 0 | 84 | 650 | 1 | 0 | 0 | 0 | 0 | 0 | 0 | 0 | 195 |
|  | Cote d'Ivoire | 832 | 0.94 | 109 | 19 | 0 | 119 | 164 | 133 | 0 | 0 | 0 | 0 | 0 | 0 | 0 | 288 |
|  | Gambia | 1271 | 9.63 | 74 | 0 | 0 | 435 | 214 | 3 | 0 | 0 | 0 | 0 | 0 | 0 | 0 | 491 |
|  | Ghana | 4389 | 2.55 | 369 | 20 | 1 | 1140 | 1540 | 225 | 0 | 1 | 9 | 0 | 0 | 0 | 0 | 1083 |
|  | Guinea | 694 | 1.79 | 37 | 0 | 0 | 109 | 269 | 14 | 0 | 0 | 0 | 0 | 0 | 0 | 0 | 249 |
|  | Guinea-Bissau | 48 | 0.49 | 32 | 1 | 0 | 0 | 0 | 0 | 0 | 0 | 0 | 0 | 0 | 0 | 0 | 15 |
|  | Liberia | 100 | 1.26 | 3 | 0 | 0 | 51 | 33 | 5 | 0 | 1 | 0 | 0 | 0 | 0 | 0 | 7 |
|  | Mali | 151 | 0.45 | 0 | 0 | 0 | 2 | 70 | 13 | 0 | 0 | 0 | 0 | 0 | 0 | 0 | 66 |
|  | Mauritania | 31 | 0.04 | 4 | 0 | 0 | 15 | 0 | 6 | 0 | 0 | 0 | 0 | 0 | 0 | 0 | 6 |
|  | Niger | 135 | 1.41 | 2 | 0 | 0 | 4 | 56 | 1 | 0 | 0 | 0 | 0 | 0 | 0 | 0 | 72 |
|  | Nigeria | 7301 | 2.73 | 257 | 2 | 0 | 2810 | 2466 | 370 | 0 | 0 | 1 | 0 | 0 | 0 | 0 | 1404 |
|  | Saint Helena | ND |  |  |  |  |  |  |  |  |  |  |  |  |  |  |  |
|  | Senegal | 4327 | 4.75 | 237 | 3 | 1 | 807 | 435 | 288 | 0 | 0 | 1 | 0 | 0 | 0 | 0 | 2465 |
|  | Sierra Leone | 56 | 0.72 | 0 | 0 | 0 | 20 | 1 | 0 | 0 | 0 | 0 | 0 | 0 | 0 | 0 | 35 |
|  | Togo | 979 | 2.47 | 25 | 5 | 0 | 435 | 413 | 47 | 0 | 0 | 0 | 0 | 0 | 0 | 0 | 176 |
|  | ***Sum*** | ***22788*** |  | ***1239*** | ***52*** | ***2*** | ***6302*** | ***6412*** | ***1353*** | ***0*** | ***2*** | ***1*** | ***0*** | ***0*** | ***0*** | ***0*** | ***7385*** |
| Central | Central African Republic | 198 | 1.28 | 9 | 0 | 0 | 8 | 69 | 1 | 0 | 0 | 0 | 0 | 0 | 0 | 0 | 111 |
|  | Chad | 49 | 0.63 | 0 | 0 | 0 | 35 | 8 | 0 | 0 | 0 | 0 | 0 | 0 | 0 | 0 | 6 |
|  | Comoros | 45 | 0.49 | 0 | 6 | 0 | 29 | 10 | 0 | 0 | 0 | 0 | 0 | 0 | 0 | 0 | 0 |
|  | Congo | 527 | 2.09 | 35 | 0 | 0 | 114 | 100 | 0 | 0 | 0 | 0 | 0 | 0 | 0 | 0 | 278 |
|  | Democratic Republic of Congo | 1437 | 1.44 | 10 | 23 | 0 | 430 | 252 | 6 | 0 | 0 | 0 | 0 | 0 | 0 | 0 | 717 |
|  | Equatorial Guinea | 200 | 1.16 | 3 | 10 | 0 | 19 | 1 | 0 | 0 | 0 | 0 | 0 | 0 | 0 | 0 | 167 |
|  | Gabon | 917 | 1.86 | 144 | 9 | 0 | 121 | 2 | 22 | 0 | 0 | 1 | 0 | 0 | 0 | 0 | 618 |
|  | Sao Tome and Principe | 10 | 0.15 | 4 | 0 | 0 | 5 | 0 | 0 | 0 | 0 | 0 | 0 | 0 | 0 | 0 | 1 |
|  | ***Sum*** | ***3383*** |  | ***205*** | ***48*** | ***0*** | ***761*** | ***442*** | ***29*** | ***0*** | ***0*** | ***1*** | ***0*** | ***0*** | ***0*** | ***0*** | ***1898*** |
| Southern | Angola | 1015 | 0.99 | 145 | 247 | 1 | 234 | 125 | 10 | 0 | 0 | 5 | 0 | 0 | 2 | 0 | 286 |
|  | Botswana | 4890 | 1.48 | 1 | 397 | 0 | 1194 | 2620 | 0 | 0 | 0 | 0 | 0 | 0 | 0 | 0 | 678 |
|  | Cameroon | 1677 | 1.34 | 19 | 12 | 0 | 318 | 1031 | 15 | 3 | 0 | 0 | 0 | 0 | 0 | 0 | 291 |
|  | Eswatini | 1072 | 1.42 | 6 | 92 | 0 | 339 | 578 | 0 | 0 | 0 | 0 | 0 | 0 | 0 | 0 | 57 |
|  | Lesotho | 251 | 0.69 | 2 | 60 | 0 | 67 | 114 | 0 | 0 | 0 | 0 | 0 | 0 | 0 | 0 | 8 |
|  | Madagascar | 806 | 1.18 | 16 | 219 | 1 | 29 | 57 | 0 | 0 | 0 | 0 | 0 | 0 | 0 | 0 | 487 |
|  | Malawi | 1167 | 1.3 | 10 | 449 | 0 | 425 | 190 | 0 | 0 | 0 | 0 | 0 | 0 | 0 | 0 | 93 |
|  | Mauritius | 6747 | 2.16 | 10 | 22 | 0 | 318 | 5411 | 0 | 0 | 0 | 0 | 0 | 0 | 0 | 0 | 986 |
|  | Mayotte | 1024 | 2.43 | 12 | 358 | 0 | 103 | 221 | 2 | 0 | 0 | 0 | 0 | 0 | 0 | 0 | 328 |
|  | Mozambique | 1379 | 0.59 | 2 | 357 | 0 | 394 | 428 | 0 | 0 | 0 | 0 | 0 | 0 | 0 | 0 | 198 |
|  | Namibia | 732 | 0.41 | 0 | 50 | 0 | 119 | 534 | 0 | 0 | 0 | 0 | 0 | 0 | 0 | 0 | 20 |
|  | South Africa | 50262 | 1.23 | 273 | 7635 | 2 | 11890 | 23816 | 15 | 0 | 0 | 18 | 0 | 0 | 0 | 2 | 6611 |
|  | Zambia | 1621 | 0.46 | 4 | 217 | 0 | 331 | 519 | 0 | 0 | 0 | 1 | 0 | 0 | 0 | 0 | 549 |
|  | Zimbabwe | 988 | 0.37 | 0 | 330 | 0 | 111 | 534 | 0 | 0 | 0 | 0 | 0 | 0 | 0 | 0 | 20 |
|  | ***Sum*** | ***73631*** |  | ***500*** | ***10445*** | ***4*** | ***15872*** | ***36178*** | ***42*** | ***3*** | ***0*** | ***24*** | ***0*** | ***0*** | ***2*** | ***0*** | ***10612*** |
| **Total** |  | **126378** |  | **3591** | **10939** | **12** | **29335** | **53888** | **1682** | **5** | **2** | **35** | **0** | **4** | **2** | **5** | **26816** |

Supplemental Table 3. Associations of the national cumulative case incidences with four individual national health indexes

| Country | Incidence /million | Mortality /million | GGHE-D/GGE (%) | CHE per capital (US$) | Physicians/1,000 | Nurses and midwives/1,000 |
| --- | --- | --- | --- | --- | --- | --- |
| Niger | 363.06 | 12.02 | 8.75 | 34.34 | 0.03 | 0.22 |
| Chad | 434.34 | 10.95 | 4.87 | 35.63 | 0.06 | 0.20 |
| Tanzania | 659.43 | 12.92 | 5.15 | 37.16 | 0.05 | 0.55 |
| Sierra Leone | 902.42 | 14.53 | 6.85 | 43.16 | 0.07 | 0.20 |
| Burkina Faso | 974.96 | 17.60 | 9.84 | 56.95 | 0.09 | 0.90 |
| Democratic Republic of Congo | 1003.26 | 14.83 | 4.34 | 22.32 | 0.36 | 1.07 |
| Nigeria | 1222.53 | 14.44 | 4.06 | 83.84 | 0.40 | 1.56 |
| Sudan | 1365.21 | 107.65 | 7.86 | 21.58 | 0.26 | 1.14 |
| Mali | 1467.76 | 32.89 | 4.96 | 40.12 | 0.12 | 0.42 |
| Liberia | 1495.47 | 55.44 | 3.66 | 112.27 | 0.05 | 1.93 |
| South Sudan | 1719.48 | 13.47 | 2.11 | 32.86 | 0.04 | 0.36 |
| Benin | 2099.62 | 12.21 | 1.6 | 35.13 | 0.06 | 0.29 |
| Madagascar | 2310.61 | 48.16 | 5.27 | 17.64 | 0.20 | 0.29 |
| Eritrea | 2765.71 | 27.96 | 2.35 | 25.36 | 0.08 | 1.44 |
| Central African Republic | 2767.45 | 20.25 | 6.42 | 43.92 | 0.07 | 0.24 |
| Guinea | 2783.10 | 33.77 | 4.49 | 44.69 | 0.22 | 0.57 |
| Angola | 2986.96 | 54.43 | 8.78 | 64.16 | 0.21 | 0.4 |
| Cote d'Ivoire | 3138.43 | 29.65 | 5.06 | 81.87 | 0.16 | 0.65 |
| Uganda | 3637.87 | 76.87 | 4.88 | 43.45 | 0.16 | 1.69 |
| Ethiopia | 4061.58 | 61.39 | 7.1 | 26.48 | 0.10 | 0.77 |
| Burundi | 4220.00 | 1.16 | 7.33 | 24.27 | 0.07 | 0.60 |
| Congo | 4222.98 | 65.15 | 8.22 | 80.51 | 0.10 | 0.93 |
| Malawi | 4369.55 | 131.63 | 5.76 | 46.56 | 0.05 | 0.70 |
| Togo | 4466.98 | 32.77 | 2.56 | 54.15 | 0.06 | 0.40 |
| Cameroon | 4482.83 | 70.72 | 2.86 | 63.79 | 0.12 | 0.19 |
| Guinea-Bissau | 4565.96 | 84.06 | 4.60 | 68.82 | 0.22 | 0.77 |
| Egypt | 4649.27 | 223.71 | 6.80 | 179.68 | 0.71 | 1.83 |
| Gambia | 4665.94 | 137.47 | 7.54 | 24.63 | 0.08 | 0.89 |
| Ghana | 5133.07 | 43.67 | 8.20 | 100.00 | 0.16 | 3.50 |
| Senegal | 5141.53 | 113.82 | 4.37 | 71.22 | 0.08 | 0.36 |
| Algeria | 6057.69 | 153.24 | 8.82 | 204.57 | 1.73 | 1.56 |
| Kenya | 6368.87 | 105.30 | 9.29 | 94.67 | 0.23 | 1.20 |
| Mozambique | 7089.31 | 68.25 | 8.15 | 44.52 | 0.08 | 0.57 |
| Rwanda | 9669.08 | 106.56 | 9.47 | 60.22 | 0.12 | 0.933 |
| Equatorial Guinea | 10227.38 | 109.26 | 5.36 | 225.57 | 0.35 | 0.27 |
| Comoros | 10885.74 | 191.21 | 4.72 | 99.04 | 0.28 | 1.59 |
| Mauritania | 13463.27 | 210.51 | 8.34 | 89.29 | 0.19 | 0.95 |
| Djibouti | 13998.29 | 168.62 | 4.20 | 87.75 | 0.26 | 0.66 |
| Lesotho | 15565.79 | 307.48 | 7.95 | 114.71 | 0.45 | 3.12 |
| Zimbabwe | 16296.95 | 351.09 | 5.21 | 62.74 | 0.19 | 2.03 |
| Zambia | 17449.78 | 203.27 | 9.25 | 75.34 | 0.30 | 1.64 |
| Gabon | 20532.05 | 128.51 | 9.59 | 233.87 | 0.59 | 2.68 |
| Sao Tome and Principe | 29547.96 | 351.81 | 13.14 | 186.1 | 0.49 | 2.15 |
| Morocco | 34119.70 | 435.10 | 7.18 | 221.11 | 0.73 | 1.39 |
| Eswatini | 62569.90 | 1187.50 | 12.30 | 279.91 | 0.14 | 2.47 |
| Namibia | 67084.69 | 1598.35 | 11.24 | 456.44 | 0.60 | 1.99 |
| South Africa | 67997.53 | 1712.95 | 15.29 | 583.67 | 0.81 | 5.01 |
| Tunisia | 93343.33 | 2381.25 | 12.40 | 265.46 | 1.26 | 2.43 |
| Cape Verde | 108695.43 | 701.33 | 15.75 | 247.91 | 0.79 | 1.24 |
| Botswana | 125616.47 | 1064.52 | 14.62 | 457.46 | 0.35 | 5.02 |
| Mauritius | 242481.98 | 812.63 | 10.2 | 564.9 | 2.658 | 3.85 |
| Seychelles | 478088.39 | 1605.45 | 10.18 | 718.49 | 2.107 | 9.22 |
| ***Region*** |  |  |  |  |  |  |
| Northern | 13632.30 | 318.71 | 7.52 (Median)  7.53 (Average) | \| 192.13 (Median) \| \| --- \| \| 154.21 (Average) \| | 0.72 (Median)  0.79 (Average) | 1.48 (Median)  1.46 (Average) |
| Eastern | 3863.14 | 61.30 | 7.10 (Median)  6.66 (Average) | 43.45 (Median)  124.21 (Average) | 0.12 (Median)  0.35 (Average) | 0.93 (Median)  1.90 (Average) |
| Western | 2142.56 | 27.31 | 5.01 (Median)  6.29 (Average) | 62.89 (Median)  74.27 (Average) | 0.11 (Median)  0.18 (Average) | 0.71 (Median)  0.93 (Average) |
| Central | 1680.20 | 21.17 | 5.89 (Median)  7.08 (Average) | 89.76 (Median)  115.87 (Average) | 0.32 (Median)  0.29 (Average) | 1.00 (Median)  1.14 (Average) |
| Southern | 20635.74 | 436.76 | 8.78 (Median)  8.96 (Average) | 75.34 (Median)  207.66 (Average) | 0.21 (Median)  0.45 (Average) | 1.99 (Median)  2.09 (Average) |

GGHE-D: domestic general government health expenditure

GGE: general government expenditure

CHE: current health expenditure
